# Predicting Mendelian Disease-Causing Non-Synonymous Single Nucleotide Variants in Exome Sequencing Studies

**DOI:** 10.1371/journal.pgen.1003143

**Published:** 2013-01-17

**Authors:** Miao-Xin Li, Johnny S. H. Kwan, Su-Ying Bao, Wanling Yang, Shu-Leong Ho, Yong-Qiang Song, Pak C. Sham

**Affiliations:** 1 Department of Psychiatry, University of Hong Kong, Pokfulam, Hong Kong, Special Administrative Region, People′s Republic of China; 2 Centre for Reproduction, Development and Growth, University of Hong Kong, Pokfulam, Hong Kong, Special Administrative Region, People′s Republic of China; 3 Centre for Genomic Sciences, University of Hong Kong, Pokfulam, Hong Kong, Special Administrative Region, People′s Republic of China; 4 Department of Medicine, University of Hong Kong, Pokfulam, Hong Kong, Special Administrative Region, People′s Republic of China; 5 Department of Biochemistry, University of Hong Kong, Pokfulam, Hong Kong, Special Administrative Region, People′s Republic of China; 6 Department of Paediatrics and Adolescent Medicine, University of Hong Kong, Pokfulam, Hong Kong, Special Administrative Region, People′s Republic of China; 7 State Key Laboratory for Cognitive and Brain Sciences, University of Hong Kong, Pokfulam, Hong Kong, Special Administrative Region, People′s Republic of China; Massachusetts General Hospital, United States of America

## Abstract

Exome sequencing is becoming a standard tool for mapping Mendelian disease-causing (or pathogenic) non-synonymous single nucleotide variants (nsSNVs). Minor allele frequency (MAF) filtering approach and functional prediction methods are commonly used to identify candidate pathogenic mutations in these studies. Combining multiple functional prediction methods may increase accuracy in prediction. Here, we propose to use a logit model to combine multiple prediction methods and compute an unbiased probability of a rare variant being pathogenic. Also, for the first time we assess the predictive power of seven prediction methods (including SIFT, PolyPhen2, CONDEL, and logit) in predicting pathogenic nsSNVs from other rare variants, which reflects the situation after MAF filtering is done in exome-sequencing studies. We found that a logit model combining all or some original prediction methods outperforms other methods examined, but is unable to discriminate between autosomal dominant and autosomal recessive disease mutations. Finally, based on the predictions of the logit model, we estimate that an individual has around 5% of rare nsSNVs that are pathogenic and carries ∼22 pathogenic derived alleles at least, which if made homozygous by consanguineous marriages may lead to recessive diseases.

## Author Summary

Sequencing the coding regions of the human genome is becoming a standard approach in identifying causal genes for human Mendelian diseases. Researchers often rely on multiple functional prediction methods/tools to separate the candidate causal mutation(s) from other rare mutations in these studies. In this paper, we propose the use of a statistical model to combine prediction scores from multiple methods and to estimate the chance of a rare mutation being Mendelian disease-causing (or pathogenic). We found that our model using all or some individual prediction methods consistently outperforms other prediction methods examined and could exclude more than 55% of rare non-pathogenic mutations in an individual genome. Unfortunately, no method was able to discriminate between autosomal dominant and autosomal recessive disease mutations. In addition, based on the predictions of our model, we estimated that a person can carry ∼22 pathogenic derived alleles at least, which if present at the same position in the genome may lead to Mendelian diseases.

## Introduction

Since the first successful application of exome sequencing in finding the causal mutation for a Mendelian disease [1], many such studies have been conducted to identify other Mendelian disease-causing (or pathogenic) variants. Compared to genetic linkage studies of Mendelian diseases, exome sequencing requires a smaller number of affected individuals, who may even be unrelated. With decreasing sequencing costs, exome sequencing is becoming a standard tool for mapping causal genes for human Mendelian diseases.

The most common cause of Mendelian disease is a non-synonymous single-nucleotide variant (nsSNV) that results in a single amino acid change in the encoded protein [2]. With the large number (typically around 8,000–10,000) of nsSNVs in an individual genome and the small number of (usually affected and unrelated) individuals available, standard methods for genetic linkage and association do not work for exome sequencing studies of Mendelian diseases. In order to narrow down the list of candidate nsSNVs, most exome sequencing studies rely on a hard-filtering approach, in which the causal mutation is assumed to be rare (with minor allele frequency (MAF) ≤1%) and so polymorphisms (with MAF>1%) found in public databases (e.g., dbSNP and 1000 Genomes Project) as well as in-house control datasets are discarded [3]. Moreover, variants are rejected if they are not found in multiple cases or if they conflicts with the known disease inheritance mode. This approach has successfully reduced the number of mutations to look at in numerous studies, and several tools [3]–[5] are therefore developed to automate this process.

However, hard-filtering in exome sequencing of Mendelian diseases still leaves a large number (typically ∼100 to 1,000) of candidate nsSNVs. A method must, therefore, be used to predict which of the remaining ones have serious functional consequences and prioritize them for validation. For a comprehensive review of these methods, see Ng and Henikoff [6]. These different methods have their complementary strengths and combining multiple methods has been suggested to increase prediction accuracy [3], [6]. Recently a combined predictive model (known as CONDEL [7]) has been developed. CONDEL is based on a Weighted Average of the normalized Scores (WAS ) [7] for combining scores from different algorithms and is available in Ensembl's Variant Effect Predictor. Another combined model (known as CAROL) is based on a weighted *Z* method of each individual score [8]. As hard filtering is usually done before prediction methods are applied in exome sequencing studies of Mendelian diseases [1], [3], it is important for prediction methods to distinguish pathogenic nsSNVs from other rare variants. However, a number of individual methods (including PolyPhen2 [9]) and both CONDEL and CAROL only use common variants as negative controls for assessing their predictive performance and determining their optimal cut-offs for variant classification. Since rare and common variants in the human genome have clearly distinct properties [10], we argue that such benchmarking may not be appropriate to exome sequencing studies.

In this paper, we first proposed the use of a logit model to combine prediction scores from multiple methods and tailored it to compute an unbiased estimate of the probability of a rare nsSNV being pathogenic. Then, we assessed the performance of five popular prediction methods (HumVar-trained PolyPhen2, SIFT [11], LRT [12], MutationTaster [13], PhyloP [14]) and two combined models (CONDEL and logit) in distinguishing pathogenic nsSNVs from other rare variants. As a comparison, we also examined the predictive powers of these methods in discriminating between pathogenic and common nsSNVs using HumVar [9] as a benchmark dataset. In addition, we saw if these prediction methods could discriminate between autosomal dominant and autosomal recessive disease mutations. Furthermore, we estimated the proportion of pathogenic rare variants and total load of pathogenic derived alleles an individual carries using high coverage exome sequencing data from the HapMap project. Finally, we applied the logit prediction model to three in-house exome sequencing subjects to demonstrate its performance in real data.

For clarity, throughout this paper, being deleterious means that an nsSNV is under purifying selection; being damaging means that an nsSNV leads to a loss of protein function; and being pathogenic means that an nsSNV has an effect on a Mendelian disease phenotype.

## Results

### Performance of prediction methods in distinguishing pathogenic nsSNVs from other rare nsSNVs

**Figure 1:**
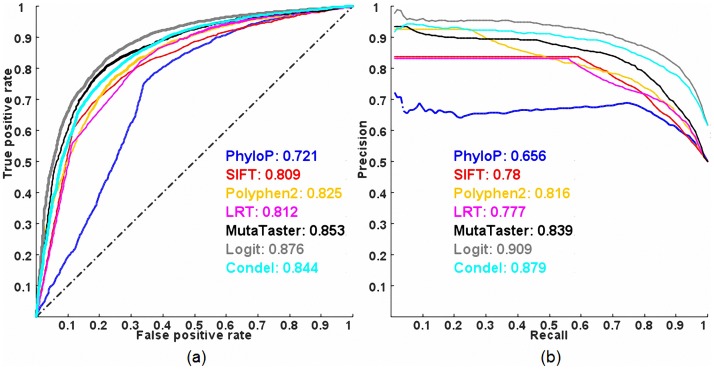
ROC and PR curves of prediction methods evaluated on the ExoVar dataset using a 10-fold cross-validation. (a) ROC and (b) PR. AUC is shown next to the name of each method.

Figure 1 shows the Receiver Operating Characteristic (ROC) and Precision-Recall (PR) curves of the five individual methods (HumVar-trained PolyPhen2 [10], SIFT [11], LRT [12], MutationTaster [13], PhyloP [14], see a description of each method in Table S1) and two combined methods (the proposed logit model and CONDEL [7], based on the scores from all five individual methods) evaluated on ExoVar (a dataset composed of pathogenic nsSNVs and nearly non-pathogenic rare nsSNVs) using a 10-fold cross-validation (see Materials and Methods). The logit model clearly outperforms all other methods in terms of the Areas Under the Curve (AUCs) of ROC and PR. The averaged maximal Matthews correlation coefficient (MCC) [15] of the logit model and CONDEL in a 10-fold cross-validation are 0.615 and 0.558 respectively. Contrary to our intuition, we found that the predictive power of CONDEL combining five individual methods is slightly inferior to that of one individual method (i.e., MutationTaster) in terms of ROC AUC, although it is better than those of all five individual methods in terms of PR AUC. Among the five individual prediction methods, MutationTaster, which considered multiple resources such as evolutionary conservation, splice-site changes and loss of protein features, outperforms the others in classifying pathogenic variants in this dataset. In contrast, PhyloP, which considers only evolutionary conservation, has the smallest AUCs, indicating that information other than evolutionary conservation is also important in classifying pathogenic nsSNVs. Nonetheless, these results suggest that the logit model is able to take advantage of the complementarily between predictions of different individual methods (which are only weakly and moderately correlated, see Figure S1) to achieve a better prediction power.

**Figure 2:**
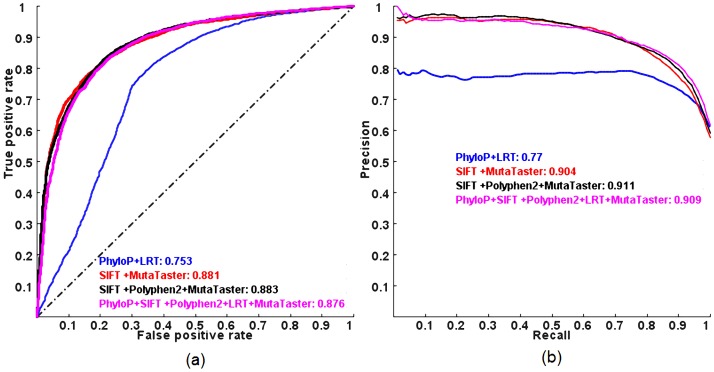
ROC and PR curves of combining a subset of the five individual methods in a logit model evaluated on the ExoVar dataset using a 10-fold cross-validation. (a) ROC and (b) PR. AUC is shown next to the name of each method.

In addition, we investigated whether combining a subset of the five individual methods in the logit model has similar predictive power compared to combining all five methods using the same validation procedure. Interestingly, we found that some reduced models have similar or slightly better predictive power than the full model (Figure 2 and Table S2). Among all possible combinations, a logit model using the scores from SIFT, Polyphen2 and MutationTaster performs the best, in terms of ROC AUC and that using the scores from SIFT and MutationTaster only has similar performance to the full model, in terms of PR AUC (so later, we will use this model in estimating the proportion of pathogenic rare nsSNVs and the total load of pathogenic derived alleles per individual as it requires non-missing scores for two methods only). In contrast, combining PhyloP and LRT, which do not incorporate any protein specific features for prediction, has the worst performance among all possible combinations.

### Performance of prediction methods in distinguishing pathogenic nsSNVs from common nsSNVs

**Figure 3:**
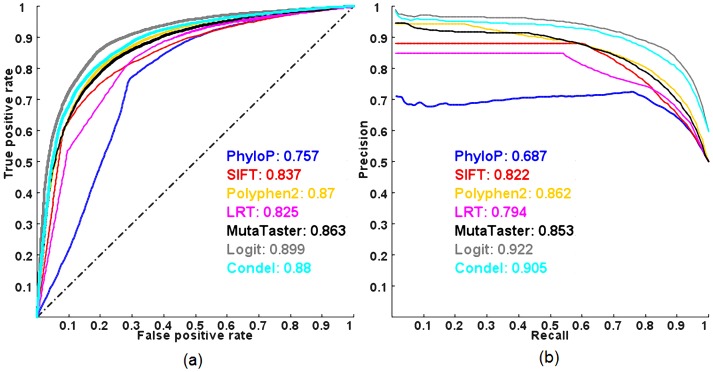
ROC and PR curves of prediction methods evaluated on the HumVar dataset using a 10-fold cross-validation. (a) ROC and (b) PR. AUC is shown next to the name of each method.

We also examined the predictive powers of the methods in discriminating between pathogenic and common nsSNVs evaluated on HumVar (a popular benchmark dataset composed of pathogenic and common nsSNVs) using a 10-fold cross-validation. Of note, the HumVar dataset was also used by PolyPhen2 and CONDEL to benchmark their prediction models for pathogenic nsSNVs. Figure 3 shows the ROC and PR curves. All combined models outperform the individual methods in terms of ROC and PR AUC, but the predictive power of a logit model is still better than that of CONDEL. The averaged maximal MCC of the logit model and CONDEL in a 10-fold cross-validation are 0.664 and 0.616 respectively. More importantly, all methods have less predictive power, in terms of ROC and PR AUC, in distinguishing pathogenic nsSNVs from other rare nsSNVs than from common nsSNVs.

### Performance of prediction methods in discriminating between autosomal dominant and autosomal recessive disease nsSNVs

**Figure 4:**
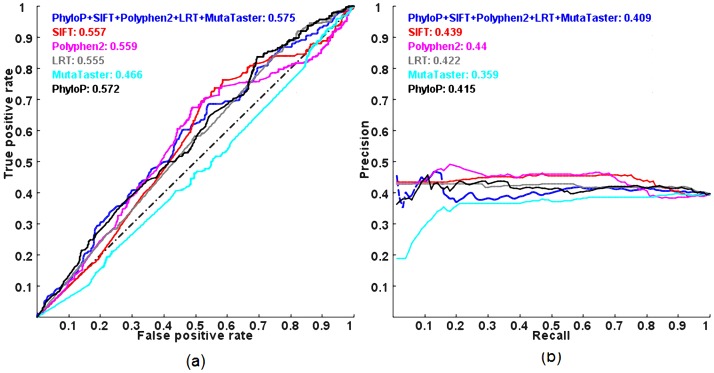
ROC and PR curves of prediction methods evaluated on the DomRec dataset using a 3-fold cross-validation. (a) ROC and (b) PR. AUC is shown next to the name of each method.

Figure 4 shows the ROC and PR curves of the methods evaluated on a dataset composed of autosomal dominant and autosomal recessive disease-causing nsSNVs, DomRec, using a 3-fold cross-validation. Although there is a significant difference in prediction scores between the two classess of disease mutations (with the largest difference observed in PhyloP, Mann-Whitney *U* test p-value = 4.56×10^−4^) (see Table 1), no method can confidently discriminate between the two classes of mutations (ROC and PR AUCs of all methods ≈0.5, i.e., random prediction) (Figure 4). These results suggest that there is not enough information to characterize autosomal dominant and recessive disease mutations in these prediction tools.

**Table 1:** Mann–Whitney *U* test *p* values for the difference in prediction scores between autosomal dominant and autosomal recessive disease-causing mutations.

	Mean scores		
Prediction score	Autosomal dominant	Autosomal recessive	Mann-Whitney *U* test p-value
PhyloP	0.968	0.929	4.56×10^−4^
SIFT	0.947	0.930	1.40×10^−3^
PolyPhen2	0.808	0.784	0.018
LRT	0.985	0.962	1.80×10^−3^
MutationTaster	0.864	0.842	7.20×10^−3^

### Estimation of the proportion of pathogenic rare nsSNVs and the total load of pathogenic derived alleles per individual

To calculate an unbiased (posterior) probability of a rare nsSNV being pathogenic in a logit model (See Materials and Methods), we need to estimate the prior probability, i.e., the proportion of pathogenic rare nsSNVs in an individual genome. Therefore, we downloaded the high coverage exome sequencing data of 8 HapMap individuals [1] and estimated the proportion of pathogenic rare nsSNVs in each individual to get an estimate of the true prior with adjustment for sensitivity and specificity of the prediction (See Materials and Methods). Also, we obtained the total load of pathogenic derived alleles (See Materials and Methods). We used a reduced logit model that combines the scores from SIFT and MutationTaster (with 80.2% averaged sensitivity and 82.8% averaged specificity ) since it has similar performance to the full model (in terms of PR AUC) and allows nsSNV with missing scores for any other three methods to be used. The proportion of predicted pathogenic rare nsSNVs ranged from 17% to 24% at individual genomes. After the adjustment of sensitivity and specificity, the true proportion of rare nsSNVs being pathogenic per individual genome is estimated to be around 0.6–12.2%, with a mean of 5%. Consequently, the total load of pathogenic derived alleles per individual genome varies from 3 to 51, with a mean of 22 (Table 2).

**Table 2:** The proportion of pathogenic rare nsSNVs and total load of pathogenic derived alleles in 8 HapMap subjects with high coverage sequencing data.

Population	Individual	Number of rare nsSNVs used^a^	% of rare variants predicted to be pathogenic	% of rare nsSNVs truly pathogenic	Total load of pathogenic derived alleles (95% CI)^b^
Caucasian	NA12156	384	20.3	5.5	21 (5, 36)
	NA12878	426	24.4	12.0	51 (33, 68)
Japanese	NA18956	356	19.1	3.6	13 (0, 26)
Chinese	NA18555	424	21.2	6.9	29 (12, 46)
African	NA18517	660	17.1	0.4	3 (0, 28)
	NA18507	623	20.9	6.4	40 (14, 64)
	NA19129	629	17.5	1.0	6 (0, 31)
	NA19240	688	18.3	2.3	16 (0, 42)

a The nsSNVs with missing scores at SIFT and/or MutationTaster were not used in the estimation.

b the 95% confidence interval was derived empirically from randomly repeating 10-fold cross-validation 200 times.

Based on the estimated average load of pathogenic derived alleles per individual (which is around 22 at least), we calculated the expected load of homozygous pathogenic variants in an offspring from consanguineous mating. Given the theoretical inbreeding coefficient (*F*) of a child of a consanguineous union [16], the corresponding number of homozygous pathogenic variants in the child equals *F•N*/2, where *N* is the total load of pathogenic derived alleles in each common ancestor of the consanguineous couple (Table 3). For example, brother–sister marriages, which constitute 20–30% of all marriages in Roman Egypt [17], would produce offspring homozygous for the pathogenic derived alleles at 3 loci. The offspring of first cousins with no family history of Mendelian diseases would theoretically have 1.4 homozygous pathogenic variants. However, in reality, there is a large variability in the values of *F* for the children of a given relationship. The offspring of first cousins can have a value of *F* as little as 3% or as much as 12% [18], which corresponds to 0.3–1.3 homozygous pathogenic variants in an individual genome. Therefore, for counseling purposes, it would be important for genetic counselors to obtain the actual proportion of genome that the consanguineous couple shares by descent from common ancestors, say by genome-wide genotyping using commercial arrays, when assessing the risk to the offspring of a couple seeking information.

**Table 3:** Theoretical inbreeding coefficient (*F*) and corresponding number of homozygous pathogenic variants in the children of various relationships, given that on average each individual carries 22 pathogenic derived alleles.

Relationship	Inbreeding coefficient, *F*	Expected number of homozygous pathogenic variants in children
Siblings	1/4	2.8
Half siblings	1/8	1.4
Uncle-niece, aunt-nephew	1/8	1.4
First cousins	1/16	0.7
First cousins once removed	1/32	0.3
Second cousins	1/64	0.2
Double first cousins	1/8	1.4
Double second cousins	1/32	0.3

### Relationship between prior and posterior probabilities of a rare nsSNV being pathogenic

**Figure 5:**
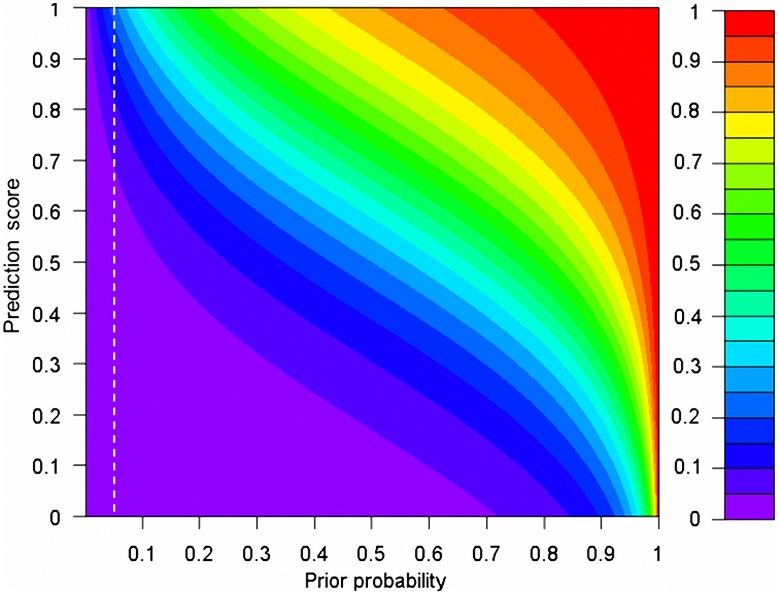
The relationship between prior and posterior probabilities of a rare nsSNV being pathogenic, given the prediction scores from SIFT, PolyPhen2, and MutationTaster. The white dashed lines indicate the estimated range of the prior (5%). We assume that there is no difference in prediction scores from the three methods for the same variant. The *α*, *β*_SIFT_, *β*_Polyphen2_ and *β*_MutationTaster_ in a selected sample evaluated in the ExoVar dataset are used in the calculation of posteriors (See Eq. 2 and 3 in Materials and Methods) and take the values of −3.53, 1.64, 1.48, and 2.47 respectively. The prior and posterior are equivalent to the quantity *P*_disease_ in an individual genome in Eq. 3 and P(*Y* = 1|*X*) in Eq. 2 respectively.

We found that, on average, around 5% of rare nsSNVs an individual carries are pathogenic (i.e., the prior probability of a rare nsSNV being pathogenic ≈5%), but this does not mean that every rare nsSNV has the same (posterior) probability of being pathogenic given the scores from individual prediction methods. Figure 5 illustrates how both prior probability and prediction scores determine the posterior probability of a rare nsSNV being pathogenic. When the prior is large (>0.9), moderate prediction scores (∼0.6) can already lead to a posterior as large as 0.8. But when the prior is small (∼0), only a small posterior is obtained, regardless of the prediction scores. Within our estimated range of the prior, the posteriors range from 6.8×10^−4^ to 0.20 when the prediction scores are increased from 0 to 1. So when all prediction scores of an nsSNV reach their maximal values of 1, the probability that the variant is pathogenic is only around 20%, indicating that a logit model alone still cannot declare a variant as pathogenic even with the strong supports from several individual methods. So additional information (like regions shared among multiple affected family members) is needed to confidently isolate the causal variant(s) [3]. In contrast, when all prediction scores of an nsSNV are close to their minimal values of 0, such probability is close to 0, indicating that a logit model is confident to declare a variant as non-pathogenic even with no or little support from individual prediction methods.

### Application of prediction methods to exome-sequencing studies

We applied the ExoVar-trained logit model using SIFT, PolyPhen2 and MutationTaster to prioritize nsSNVs in 3 Mendelian-disease patients with in-house exome sequencing data. Table 4 shows the numbers and percentages of nsSNVs removed by the hard-filtering approach and functional prediction using the logit model in these patients. Although hard-filtering (MAF>1% in dbSNP, HapMap and 1000 Genomes) can exclude ∼7,000 (more than 80% of) nsSNVs, there are still ∼1,000 variants left for examination in each patient. Prediction using different logit models can further exclude more than 500 (55% of) nsSNVs. Of note, the causal mutations in these patients (i.e., c.1528G>C of *TGM6* for spinocerebellar ataxia patients [19] in patients 1 and 2 as well as compound heterozygosity for *IL10RA* mutations: c.251C>T and c.301C>T [20] in patient 3 with neonatal onset Crohn's disease) were all predicted to be pathogenic by the logit model.

**Table 4:** The numbers and proportion of nsSNVs removed by hard-filtering and functional prediction by the logit model in 3 Mendelian-disease patients with in-house exome sequencing data.

	Patient 1^a^	Patient 2^a^	Patient 3^b^
Missense, stop-loss and stop-gain variants	8,383	8,563	7,969
MAF>1%	−7,398 (−88.3%)	−7,552(−88.2%)	−6,751(−84.7%)
	= 985	= 1,011	= 1,218
Predicted to be non-pathogenic	−569 (−57.8%)	−583 (−57.7%)	−702 (−57.6%)
	= 416^c^	= 428^c^	= 516^c^

a Related cases with autosomal dominant spinocerebellar ataxia.

b Case with neonatal-onset Crohn's disease.

c nsSNVs in which prediction is unavailable due to missing scores.

## Discussion

In this paper, we propose to use a logit model to combine multiple prediction methods to increase the performance in predicting pathogenic nsSNVs and to compute an unbiased (posterior) probability of an nsSNV being pathogenic in exome sequencing after hard-filtering. Also, we examine the predictive power of five popular prediction methods (PolyPhen2, SIFT, LRT, MutationTaster, PhyloP) and two combined models (CONDEL and logit) in discriminating between pathogenic nsSNVs and other rare nsSNVs (which is strictly relevant to exome sequencing studies). Contrary to our intuition, we found that the combined approach CONDEL is not necessarily better than individual methods, as demonstrated by its lower ROC AUC compared to that of MutationTaster. However, the logit model using multiple individual methods consistently outperforms other methods examined and the model combining SIFT and MutationTaster has comparable or even slightly better performance than that combining all of the five individual methods. Unfortunately, no method is able to discriminate between autosomal dominant and autosomal recessive disease mutations. Finally, based on the predictions of the logit model, we estimate that an individual has around 5% of rare nsSNVs being pathogenic and carries at least ∼22 pathogenic derived alleles, which if made homozygous by consanguineous marriages may lead to recessive diseases.

We found that prediction methods are less powerful in predicting pathogenic variants from other rare variants than from common variants. This is consistent with the fact that most rare alleles (no matter whether they are pathogenic or not) of nsSNVs are subject to strong purifying selection and therefore have similar structural and functional properties, whereas common nsSNVs (in which the minor alleles are also found at high frequency in populations) are subject to weak purifying selection and so have nearly different properties compared to rare nsSNVs. Thus, as expected, it is more difficult to separate pathogenic mutations from other rare nsSNVs than from common nsSNVs using prediction methods.

There is a significant difference in PhyloP conservation scores between dominant and recessive disease mutations. This may be because dominant disease genes are more conserved than recessive disease genes which can be “hidden” from purifying selection while heterozygous [21]. However, none of the prediction methods we examined, which mainly use the genomic features at variant level, are able to distinguish autosomal dominant mutations from autosomal recessive disease-causing mutations. Presumably, the genomic features at gene level may help distinguish them. Some studies have analyzed the difference in functional classification of the two classes of disease mutations and found that mutations in genes coding for enzymes and transporters are most likely to cause recessive diseases, whereas mutations in transcription regulators, structural molecules, nucleic acid binding genes and signal transducers have a higher chance to cause dominant diseases [22], [23]. Also, genes involved in recessive diseases have less conserved paralogs than dominant disease genes [21], [23], as recessive diseases are often caused by loss-of-function mutations [22], [24] (which create a defective protein product with little or no biologic activity, and/or interfere with the normal expression of the gene). If a close paralog of a recessive disease gene is present, the paralog is likely to compensate for the loss of function due to a mutated recessive disease gene and so the disease is not observed [25]. On the contrary, dominant diseases are usually caused by gain-of-function mutations (which confer a new activity on the gene product, or lead to its inappropriate spatial and temporal expression) and so the presence of wild-type proteins encoded by functionally similar paralogs may not suppress the new functions acquired by the mutant proteins [23].

We found that the correlations among the scores from several complementary prediction methods are mostly weak to moderate (see Figure S1). This can occur for two possible reasons. First, the set of species used by one method for measuring conservation may be significantly different from those used by another, and thus this may lead to a big difference between the prediction scores calculated by the different methods for the same site. Second, the set of perfectly conserved sites used for training by one method may also be different from the ones used by others due to the variation in sequence alignments adopted by each method. Nevertheless, to our knowledge, MutationTaster uses the largest amount of resources for training and this may explain its excellent predictive performance among the five individual methods examined. But some redundancy among prediction scores from multiple methods also explains why combining a subset of the five individual methods (i.e., PolyPhen2, SIFT, and MutationTaster) has similar predictive performance to combine all five individual methods in a logit model.

We note that our estimates of the number of pathogenic alleles per individual from the human genome data are higher than those from the data on consanguineous marriages (which suggest a much smaller number, usually less than 10 [26]–[30]). But comparison of our estimates with those from inbreeding studies is difficult since we use totally different method and data. A similar situation has also occurred in quantifying the number of lethal equivalents per individual, in which inbreeding studies suggest that each individual carries 2-6 lethal equivalents [31], [32] whereas Kondrashov [33] found the number could be as high as 100. Anyway, estimation from inbreeding studies typically relies on an implicit assumption that all recessive alleles are completely penetrant and expressive, but examples that violate this assumption have recently been found. For example, the presence of a dominant modifier DFNM1 leads to normal hearing in an individual homozygous for the DFNB26 mutation [34]; high expression of actin-binding protein plastin 3 (PLS3) protects individuals carrying homozygous SMN1 deletions from developing spinal muscular atrophy (SMA) [35]; and among the two siblings affected by autosomal recessive polycystic kidney disease (ARPCKD), one died at 18 hr but the other still had no symptom when presented at 16 [36]. So the numbers from inbreeding studies are likely to be an underestimate. However, mapping errors may also inflate our estimates. For example, it was found that sequencing variants in the inactive gene copy of CDC27 gene (i.e., pseudogene) were wrongly mapped to the active gene copy of CDC27 [37] and we observed that the active gene copy of CDC27 had as many as 11 nsSNVs at 2 out of 8 HapMap subjects examined. It is likely that some of these nsSNVs actually came from CDC27 pseudogene(s) and that can therefore inflate our estimates. But missing scores at sequencing variants could, on the other hand, deflate our estimates. Around 13–17% of rare nsSNVs in an individual have missing scores at SIFT and/or MutationTaster and so pathogenic alleles in these variants cannot be counted.

We also observed a marked variability in our estimates of the number of pathogenic alleles per individual. The statistical fluctuation of specificity is the major reason for the large variability. As shown in Table 2, a standard error of 1.3% in specificity can already lead to a standard error of 1.9% in the estimated proportion of pathogenic rare variants and finally results in a standard error of ∼10–15 in the estimated total load of pathogenic rare variants.

We showed that, after MAF filtering, the prior (i.e., the proportion of variants left being pathogenic) is low (which is around 5% and leads to a posterior probability of ∼20% at most) and so it is still difficult for prediction methods to pinpoint the pathogenic mutation(s) in exome sequencing studies of Mendelian diseases. One way to increase the prior is to use additional information, including genomic regions shared by multiple affected family members and known biological pathways, to reduce the number of candidate pathogenic variants and therefore we have implemented these functions in KGGSeq. We also found that even low prediction scores can lead to a posterior that can help exclude non-pathogenic variants. Using the exome sequencing data of three patients with Mendelian diseases, we observed that a logit model could exclude more than 55% of rare nsSNVs. Moreover, these posterior probabilities can be used as weights of the nsSNVs for other analyses.

## Materials and Methods

### Benchmark datasets

#### ExoVar

This dataset is composed of 5,340 alleles with known effects on the molecular function causing human Mendelian diseases from the UniProt database, which are treated as positive control variants, and 4,752 rare (alternative/derived allele frequency <1%) nsSNVs with at least one homozygous genotype for the alternative/derived allele in the 1000 Genomes Project, which are treated as negative control variants. This dataset can be downloaded from our KGGSeq website (see Data Access). It is used for evaluating the performance of prediction methods in distinguishing pathogenic nsSNVs from other rare variants. This benchmark dataset can be downloaded at http://statgenpro.psychiatry.hku.hk/limx/kggseq/download/ExoVar.xls.

#### HumVar

This dataset was prepared by the PolyPhen2 team for benchmarking their program and is available online (see Data Access). It consists of 22,196 human disease-causing or loss of activity/function mutations (except cancer mutations) present in the UniProtKB database, which are treated as positive control variants, together with 21,151 common (MAF>1%) nsSNVs with no reported disease association, which are treated as negative control variants. Of note, HumVar was used by PolyPhen2 and CONDEL for benchmarking models used for predicting pathogenic nsSNVs.

#### DomRec

To examine the performance of predictive models in discriminating between autosomal dominant and autosomal recessive disease mutations, we retrieved from Galaxy [38], [39] 253 nsSNVs causing only autosomal dominant diseases and 389 nsSNVs causing only autosomal recessive diseases.

### Functional prediction scores

We obtained, from dbNSFP database v2.0 [40], the prediction scores from four prediction algorithms (SIFT, HumVar-trained Polyphen2, LRT and MutationTaster) and a conservation score (PhyloP) for each nsSNV in the human genome (hg19). Some methods (e.g. MutationTaster) reported predictions for alternative (or non-reference) alleles while some (e.g. PolyPhen2) conducted predictions for derived (or non-ancestral) alleles. To avoid inconsistency in predictions, we removed variants whose alternative alleles are not derived alleles. We also downloaded the CONDEL perl script from the authors' website (see Data Access) and used it to compute a CONDEL WAS score for each nsSNV based on the scores from all five individual methods. For all methods, the scores were standardized to range from 0 to 1 and, in general, the larger the score, the higher the probability of causing diseases.

### Exome sequencing datasets

#### HapMap

We downloaded the protein coding SNVs of 8 HapMap individuals with high coverage exome sequencing data [1] (see Data Access). They are NA12156 and NA12878from Caucasians, NA18956 from Japanese (Asian), NA18555 from Chinese (Asian), as well as NA18517, NA18507, NA19129 and NA19240 from Africans.

#### In-house

Two samples came from an autosomal dominant spinocerebellar ataxia pedigree [19] and another from a neonatal-onset Crohn's disease pedigree [20]. They were collected in Hong Kong with Institutional Review Board approval and were sequenced by the Illumina Genome Analyzer II platform at deCODE Genetics. The paired-end 76-bp short reads from Illumina Genome Analyzer II were aligned and mapped onto the UCSC human reference genome (hg18), by Burrows-Wheeler Aligner [41]. Duplicated reads were marked by Picard (see Data Access). The Genome analysis toolkit (GATK) [42] was then used to recalibrate the alignments and to call SNVs (by UnifiedGenotyper).

### Logit model for combining individual prediction tools

Given a vector, *X*, of prediction scores from multiple prediction methods for a particular nsSNV, the (posterior) probability that the nsSNV is pathogenic (*Y* = 1) is given by:
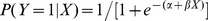
(1)where *α* and *β* are, respectively, the constant and vector of coefficients of *X* from logistic regression on a population (or random sample) of pathogenic (positive control) and other (negative control) variants. However, when the sample (benchmark dataset) is selected, the probability in Eq. 1 would be biased. An unbiased estimate of (posterior) probability can be obtained by (see Text S1):
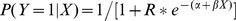
(2)where *R* is the odds of a variant being pathogenic in the selected sample divided by that in the population (i.e., an individual genome), i.e.,
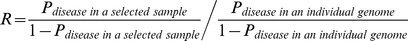
(3)where *P*_disease_ denotes the proportion of pathogenic nsSNVs. Note that the value of *R* is the same for all variants and affects only the probability calculated, but not variant classification. Also, in our case, *P*_disease_ in a selected sample is simply the proportion of positive control variants among all variants included in the benchmark dataset. We will demonstrate how *P*_disease_ in an individual genome can be obtained in the section below.

### Assessment of predictive power

We evaluated the performance of five individual methods (SIFT, HumVar-trained Polyphen2, LRT, MutationTaster, and PhyloP) and two combined methods (CONDEL WAS and logit) in discriminating between

1. pathogenic nsSNVs and other rare variants using a 10-fold cross-validation on the ExoVar dataset;
2. pathogenic nsSNVs and common variants using a 10-fold cross-validation on the HumVar dataset, and;
3. autosomal dominant and autosomal recessive disease-causing nsSNVs using a 3-fold cross-validation on the DomRec dataset.

The parameters *α* and *β* in the logit model, as well as the complementary cumulative distributions and optimal cutoffs (that maximizes MCC) for each individual prediction method required by CONDEL were obtained from the training dataset in each fold in a *K*-fold cross-validation. When we validated the trained models in a test dataset, the probability cutoff was increased from 0 to 1 and the corresponding true positive prediction rate (sensitivity) and true negative prediction rate (specificity) of different prediction methods were recorded. We built the ROC and PR curves and reported the MCC [15], ROC AUC and PR AUC of each method evaluated on each benchmark dataset. The program AUCCalculator [43] was used to calculate the AUCs. The sensitivities and specificities from *K* folds in a *K*-fold cross-validation were averaged for ROC and PR curve plotting. For the logit model, the quantity *R* in Eq. 3 is assumed to be 1 as this affects only the biasness of the probability calculated but not classification. Furthermore, we evaluated the effect of using a subset instead of all five individual methods in the logit model on the power in distinguishing pathogenic nsSNVs from other rare variants using a 10-fold cross-validation on the ExoVar dataset.

### Estimation of the proportion of pathogenic rare nsSNVs and the total load of pathogenic derived alleles per individual

To obtain an estimated range of *P*_disease_, i.e., the proportion of pathogenic rare nsSNVs in an individual genome, in Eq. 3, we need to get an estimated range of P^*^_disease_, i.e., the proportion of predicted pathogenic rare nsSNVs in an individual genome. To estimate P^*^_disease_, we downloaded the high coverage exome sequencing data of 8 HapMap individuals and removed common nsSNVs annotated in dbSNP and 1000 Genomes using KGGSeq. Then, prediction was done in these filtered datasets using a logit model (with a cutoff that maximizes the MCC evaluated on the ExoVar dataset) to obtain P^*^_disease_ for each of the individuals. Given the specificity and sensitivity of the prediction model used, we have
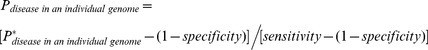
(4)Since the number of homozygous genotypes for the derived allele in an individual is small compared to that of heterozygous genotypes, result from the prediction and estimation on all genotypes is similar to that on heterozygous genotypes only. So, for simplicity, we reported the latter results. That also simplifies our calculation of the total load of pathogenic derived alleles in each individual presented as the value just equals the number of pathogenic rare variants.

To quantify the variability in the estimates of the total load of pathogenic derived alleles per individual, we randomly repeated the 10-fold cross validation 200 times. Each round of 10-fold cross validation generates a pair of averaged sensitivity and specificity values and this pair of values is then used to calculate *P*_disease_ according to equation (4) and the total load of pathogenic derived alleles. We obtained the 95% confidence interval (CI) of the total load of pathogenic derived alleles by taking the 2.5% and 97.5% quantiles in the distribution of 200 such estimates.

### Implementation

We implemented the logit model as one of important functions in our KGGseq software package, which was designed to conduct knowledge-based downstream analyses in sequencing studies. KGGSeq also gives a posterior probability of being pathogenic for each nsSNV by combining the five individual prediction scores available in the dbNSFP database v2.0 [40]. These features should facilitate the ranking and/or filtering of nsSNVs in exome sequencing studies of Mendelian diseases.

### Application to exome sequencing data

We applied the logit model to the in-house exome sequencing data of three patients. KGGSeq was used to exclude variants and genotypes with low quality (read coverage ≤4×, Phred-scaled base sequencing quality ≤50, and Phred-scaled genotype calling quality score ≤20), map variants onto the reference genome (hg18), extract nsSNVs as well as remove known common nsSNVs (annotated in dbSNP and 1000 Genomes). Finally, for each of the remaining rare nsSNVs, KGGSeq assigned a logit prediction score, a posterior probability of being pathogenic and decided whether it is pathogenic or not using a cutoff that maximizes the MCC in ExoVar dataset.

### Data access

The URLs for data presented herein are as follows:

KGGseq software tool, http://statgenpro.psychiatry.hku.hk/kggseq/

ExoVar dataset, http://statgenpro.psychiatry.hku.hk/limx/kggseq/download/ExoVar.xls

dbNSFP, http://sites.google.com/site/jpopgen/dbNSFP

Galaxy, http://main.g2.bx.psu.edu/library

Polyphen2HumVar dataset, http://www.nature.com/nmeth/journal/v7/n4/suppinfo/nmeth0410-248_S1.html

Online Mendelian Inheritance in Man, http://www.ncbi.nlm.nih.gov/Omim

1000 Genomes Project data, http://www.sph.umich.edu/csg/abecasis/MACH/download/

Polyphen-2 website, http://genetics.bwh.harvard.edu/pph2/

UniProt website, http://www.uniprot.org/

Picard website, http://picard.sourceforge.net/;

High coverage exome sequencing data of 8 HapMap individuals, http://krishna.gs.washington.edu/12_exomes/;

CONDEL, http://bg.upf.edu/condel/home

## Supporting Information

Figure S1 Spearman's rank correlation matrix for the scores from the five individual algorithms (SIFT, HumVar-trained Polyphen2, LRT, MutationTaster, and PhyloP) in HumVar's a) positive control variants, b) negative control variants; ExoVar's c) positive controls, d) negative controls.
(DOC)

Table S1 Descriptions of individual prediction methods evaluated.
(DOC)

Table S2 The ROC and PR AUCs of the logit model using a subset of the five individual algorithms evaluated on the ExoVar dataset using a ten-fold cross-validation.
(DOC)

Text S1 Computing the unbiased (posterior) probability from logistic regression trained on a selected sample.
(DOCX)
