# Supplementary material for: Predicting Mendelian Disease-Causing Non-Synonymous Single Nucleotide Variants in Exome Sequencing Studies
Source: PLoS Genet. 2013 Jan 17;9(1):e1003143. doi: 10.1371/journal.pgen.1003143 (PMC3547823; doi:10.1371/journal.pgen.1003143)
Supplement: Table S1 — Descriptions of individual prediction methods evaluated. (DOC) [file pgen.1003143.s002.doc]

| **Method** | **Description** |
| --- | --- |
| PhyloP | PhyloP estimates the evolutional conservation at each variant from multiple alignments of placental mammal genomes to the human genome based on a phylogenetic hidden Markov model. In the present study, the PhyloPscores were standardized by the formula *S-S_min_*/(*S_max_-S_min_*), where the *S_min_* and *S_max_* are the minimum and maximum of the observed PhyloP scores. |
| SIFT | SIFT use the 'Sorting Tolerant From Intolerant' (SIFT) algorithm to predict whether a single amino acid substitution affects protein function or not, based on the assumption that important amino acids in a protein sequence should be conserved throughout evolution and substitutions at highly conserved sites are expected to affect protein function. Originally, a small score, *S*, indicates a high chance for a substitutionto damage the protein function. Here, we use an adjusted score, 1 –*S*. |
| Polyphen2 | Polyphen2 predicts the possible impact of an amino acid substitution on the structure and function of a human protein using straightforward physical and comparative considerations by an iterative greedy algorithm. In the present study, we use the original scores generated by the HumVar (instead of HumDiv) trained model as it is preferred for the diagnosis of Mendelian diseases. The scores range from 0 to 1. A substitution with larger score has a higher possibility to damage the protein function. |
| LRT | LRT employed a likelihood ratio test to assess variant deleteriousness based on a comparative genomics data set of 32 vertebrate species. The identified deleterious mutations could disrupt highly conserved amino acids within protein-coding sequences, which are likely to be unconditionally deleterious.The scores range from 0 to 1. A larger score indicates a larger deleterious effect. |
| MutationTaster | MutationTasterassesses the impact of the disease-causing potential of a sequence variant by a naive Bayes classifier using multiple resources such as evolutionary conservation, splice-site changes, loss of protein features and changes that might affect mRNA level. The scores range from 0 to 1. The larger score suggests a higher probability to cause a human disease. |
