# Supplementary material for: Predicting Mendelian Disease-Causing Non-Synonymous Single Nucleotide Variants in Exome Sequencing Studies
Source: PLoS Genet. 2013 Jan 17;9(1):e1003143. doi: 10.1371/journal.pgen.1003143 (PMC3547823; doi:10.1371/journal.pgen.1003143)
Supplement: Table S2 — The ROC and PR AUCs of the logit model using a subset of the five individual algorithms evaluated on the ExoVar dataset using a ten-fold cross-validation. (DOC) [file pgen.1003143.s003.doc]

| **Combination** | **ROC** | | **PR** | |
| --- | --- | --- | --- | --- |
|  | AUC | Rank | AUC | Rank |
| SIFT, Polyphen2, MutationTaster | 0.8834 | 1 | 0.9131 | 2 |
| SIFT, MutationTaster | 0.8818 | 2 | 0.9014 | 6 |
| PhyloP, SIFT, Polyphen2, MutationTaster | 0.8800 | 3 | 0.9010 | 7 |
| SIFT, Polyphen2, LRT, MutationTaster | 0.8797 | 4 | 0.9177 | 1 |
| SIFT, LRT, MutationTaster | 0.8783 | 5 | 0.9129 | 3 |
| PhyloP, SIFT, MutationTaster | 0.8769 | 6 | 0.8912 | 11 |
| Polyphen2, MutationTaster | 0.8761 | 7 | 0.8998 | 8 |
| PhyloP, SIFT, Polyphen2, LRT, MutationTaster | 0.8759 | 8 | 0.9078 | 5 |
| PhyloP, SIFT, LRT, MutationTaster | 0.8712 | 9 | 0.8993 | 9 |
| PhyloP, Polyphen2, MutationTaster | 0.8707 | 10 | 0.8848 | 13 |
| Polyphen2, LRT, MutationTaster | 0.8692 | 11 | 0.9096 | 4 |
| PhyloP, Polyphen2, LRT, MutationTaster | 0.8641 | 12 | 0.8935 | 10 |
| PhyloP, MutationTaster | 0.8549 | 13 | 0.8543 | 21 |
| SIFT, Polyphen2, LRT | 0.8508 | 15 | 0.8905 | 12 |
| LRT, MutationTaster | 0.8508 | 14 | 0.8744 | 16 |
| PhyloP, SIFT, Polyphen2, LRT | 0.8436 | 16 | 0.8764 | 15 |
| PhyloP, LRT, MutationTaster | 0.8423 | 17 | 0.8626 | 19 |
| SIFT, Polyphen2 | 0.8421 | 18 | 0.8704 | 17 |
| PhyloP, SIFT, Polyphen2 | 0.8409 | 19 | 0.8586 | 20 |
| SIFT, LRT | 0.8403 | 20 | 0.8786 | 14 |
| Polyphen2, LRT | 0.8321 | 21 | 0.8646 | 18 |
| PhyloP, Polyphen2, LRT | 0.8248 | 22 | 0.8527 | 22 |
| PhyloP, Polyphen2 | 0.8245 | 23 | 0.8364 | 24 |
| PhyloP, SIFT, LRT | 0.8235 | 24 | 0.8526 | 23 |
| PhyloP, SIFT | 0.8170 | 25 | 0.8199 | 25 |
| PhyloP, LRT | 0.7525 | 26 | 0.7700 | 26 |
