# Supplementary material for: Predicting Mendelian Disease-Causing Non-Synonymous Single Nucleotide Variants in Exome Sequencing Studies
Source: PLoS Genet. 2013 Jan 17;9(1):e1003143. doi: 10.1371/journal.pgen.1003143 (PMC3547823; doi:10.1371/journal.pgen.1003143)
Supplement: Text S1 — Computing the unbiased (posterior) probability from logistic regression trained on a selected sample. (DOCX) [file pgen.1003143.s004.docx]

Given a population (or random sample) *P* of positive (*Y_P_* = 1) and negative (*Y_P_* = 0) controls, a selected (i.e., non-random) sample *S* of positive (*Y_S_* = 1) and negative (*Y_S_* = 0) controls from *P*, and a vector *X* of predictor variables (i.e., prediction scores), the logit function for *X* in *S* (*X_S_*) is,

| $\ln\left[ \frac{P(Y_{S}=1\vert X_{S})}{P(Y_{S}=0\vert X_{S})} \right]=\alpha_{S}+\beta_{S}X_{S}$ | (1) |
| --- | --- |

where *α_S_* and *β_S_* are, respectively, the constant and vector of coefficients of *X* from logistic regression on *S*. Similarly, the logit function for *X* in *P* (*X_P_*) is,

| $\ln\left[ \frac{P(Y_{P}=1\vert X_{P})}{P(Y_{P}=0\vert X_{P})} \right]=\alpha_{P}+\beta_{P}X_{P}$ | (2) |
| --- | --- |

where *α_P_* and *β_P_*are, respectively, the constant and vector of coefficients of *X* from logistic regression on *P*.

Using the Bayes' theorem, the odds of being a positive control in *S* is

$\frac{P(Y_{S}=1|X_{S})}{P(Y_{S}=0|X_{S})}=\frac{P(X_{S}|Y_{S}=1)P(Y_{S}=1)}{P(X_{S})}/\frac{P(X_{S}|Y_{S}=0)P(Y_{S}=0)}{P(X_{S})}=\frac{P(X_{S}|Y_{S}=1)}{P(X_{S}|Y_{S}=0)}\frac{P(Y_{S}=1)}{P(Y_{S}=0)}$(3)

Similarly,

| $\frac{P(Y_{P}=1\vert X_{P})}{P(Y_{P}=0\vert X_{P})}=\frac{P(X_{P}\vert Y_{P}=1)}{P(X_{P}\vert Y_{P}=0)}\frac{P(Y_{P}=1)}{P(Y_{P}=0)}$ | (4) |
| --- | --- |

Since the conditional distribution of *X* given *Y* is unaffected by the sampling on *Y*, i.e.,

$P(X_{S}|Y_{S}=1)=P(X_{P}|Y_{P}=1)$ and $P(X_{S}|Y_{S}=0)=P(X_{P}|Y_{P}=0)$

From Eq.3 and 4, we have,

| $\ln[\frac{P(Y_{S}=1\vert X_{S})}{P(Y_{S}=0\vert X_{S})}]-\ln[\frac{P(Y_{P}=1\vert X_{P})}{P(Y_{P}=0\vert X_{P})}]=\ln[\frac{P(Y_{S}=1)}{P(Y_{S}=0)}]-\ln[\frac{P(Y_{P}=1)}{P(Y_{P}=0)}]$ | (5) |
| --- | --- |

Substituting Eq. 1 and 5 into Eq. 2, we get,

| $\ln\left[ \frac{P(Y_{P}=1\vert X_{P})}{P(Y_{P}=0\vert X_{P})} \right]=ln\left[ \frac{P(Y_{S}=1)}{P(Y_{S}=0)} \right]-{\ln\left[ \frac{P(Y_{P}=1)}{P(Y_{P}=0)} \right]+\alpha}_{S}+\beta_{S}X_{S}$ | (6) |
| --- | --- |

Since $P\left( Y_{P}=0 | X_{P} \right)=1-P(Y_{P}=1|X_{P})$, we obtain

| $P\left( Y_{P}=1 \vert X_{P} \right)=1/\left[ 1+Re^{-(\alpha_{S}+\beta_{S}X_{S})} \right]$ | (7) |
| --- | --- |

where$R={\frac{P(Y_{S}=1)}{P(Y_{S}=0)}}/{\frac{P(Y_{P}=1)}{P(Y_{P}=0)}}$
