# Supplementary material for: Predicting Mendelian Disease-Causing Non-Synonymous Single Nucleotide Variants in Exome Sequencing Studies
Source: PLoS Genet. 2013 Jan 17;9(1):e1003143. doi: 10.1371/journal.pgen.1003143 (PMC3547823; doi:10.1371/journal.pgen.1003143)
Supplement: Supplementary file 4 [file pgen.1003143.s001.doc]

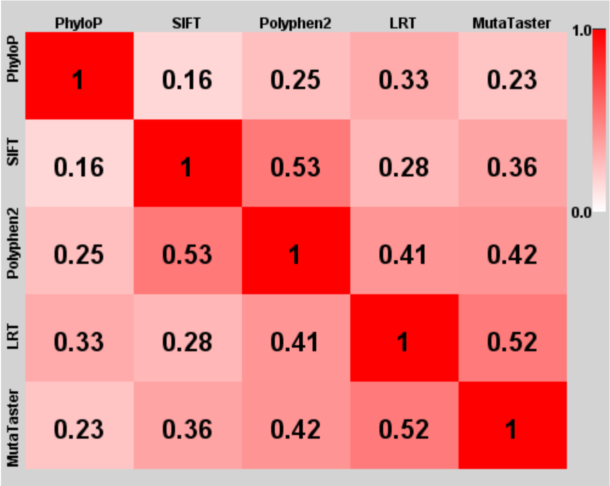
PhyloP SIFT Polyphen2 LRT MutaTaster
1.0
0.0
PhyloP 1 0.16 0.25 0.33 0.23
SIFT 0.16 1 0.53 0.28 0.36
Polyphen2 0.25 0.53 1 0.41 0.42
LRT 0.33 0.28 0.41 1 0.52
MutaTaster 0.23 0.36 0.42 0.52 1
(a)

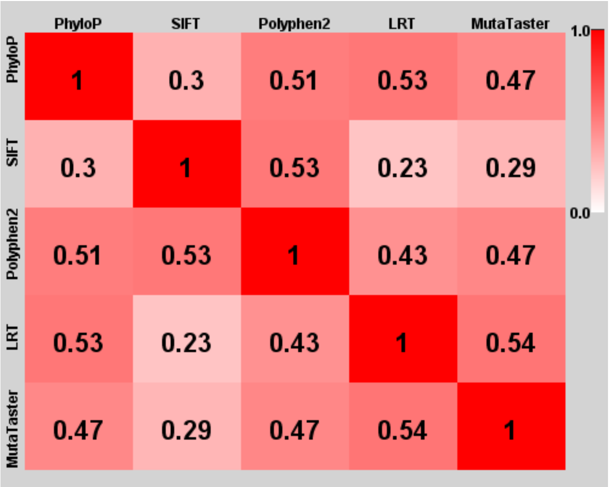
PhyloP SIFT Polyphen2 LRT MutaTaster
1.0
0.0
PhyloP 1 0.3 0.51 0.53 0.47
SIFT 0.3 1 0.53 0.23 0.29
Polyphen2 0.51 0.53 1 0.43 0.47
LRT 0.53 0.23 0.43 1 0.54
MutaTaster 0.47 0.29 0.47 0.54 1
(b)

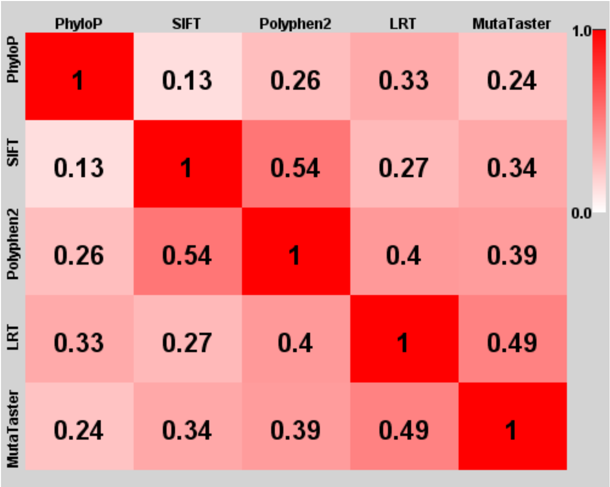
PhyloP SIFT Polyphen2 LRT MutaTaster
1.0
0.0
PhyloP 1 0.13 0.26 0.33 0.24
SIFT 0.13 1 0.54 0.27 0.34
Polyphen2 0.26 0.54 1 0.4 0.39
LRT 0.33 0.27 0.4 1 0.49
MutaTaster 0.24 0.34 0.39 0.49 1
(c)

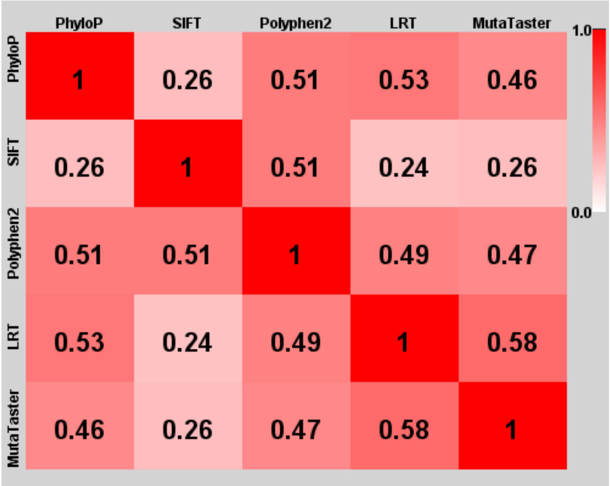
PhyloP SIFT Polyphen2 LRT MutaTaster
1.0
0.0
PhyloP 1 0.26 0.51 0.53 0.46
SIFT 0.26 1 0.51 0.24 0.26
Polyphen2 0.51 0.51 1 0.49 0.47
LRT 0.53 0.24 0.49 1 0.58
MutaTaster 0.46 0.26 0.47 0.58 1
(d)
